# Direct radiation damage to human tooth under IMRT for head and neck cancer: physicochemical evidence supporting a non-salivary mechanism for radiation-related caries

**DOI:** 10.1007/s00411-025-01180-w

**Published:** 2025-12-15

**Authors:** Toru Tamahara, Atsumu Kouketsu

**Affiliations:** 1 Division of Community Oral Health Science, Department of Community Medical Supports, Tohoku Medical Megabank Organization, Tohoku University, 2-1, Seiryomachi, Aobaku, Sendai, 980-0873 Japan; 2 Division of Oral and Maxillofacial Oncology and Surgical Sciences, Department of Disease Management Dentistry, Tohoku University Graduate School of Dentistry, Sendai, Japan

**Keywords:** Radiation-related caries, Head and neck cancer, IMRT, Collagen, Microhardness, Dental tissue

## Abstract

Radiation-related dental caries (RRC) is a rapidly progressing and treatment-resistant condition commonly observed after head and neck radiotherapy. It has traditionally been attributed to radiation-induced salivary gland dysfunction; however, RRC often develops even when appropriate oral care is provided, including fluoride application and regular hygiene practices. This clinical inconsistency suggests that salivary dysfunction alone cannot fully explain the onset and severity of RRC. The direct physicochemical effects of radiation on the organic and inorganic components of dental hard tissues remain poorly understood. In this study, extracted human third molars were subjected to a clinical IMRT protocol, using either a single high dose (20 Gy × 1) or a fractionated regimen (2 Gy/day × 35, total 70 Gy). We then evaluated radiation-induced changes in tooth structure by measuring Vickers microhardness, acid resistance (calcium elution), collagen degradation (autofluorescence), and internal dentin pH. Radiation exposure significantly altered enamel hardness: it increased after 20 Gy but decreased following 70 Gy. Calcium release increased in crown enamel (70 Gy) and root dentin (20 Gy), while decreasing in crown dentin (20 Gy). Autofluorescence imaging showed a significant reduction in dentinal collagen after fractionated exposure. Both groups exhibited an alkaline shift in dentin pH, more pronounced in the single-dose group. These findings provide physicochemical evidence for a non-salivary mechanism contributing to RRC. To effectively prevent RRC, additional strategies to reduce direct radiation damage to dental tissues may be necessary alongside conventional salivary-focused approaches.

## Introduction

Radiation-related dental caries (RRC) is a severe and progressive form of tooth decay that emerges as a late complication in patients undergoing head and neck radiotherapy. Clinically, it is characterized by a rapid onset, multifocal presentation, and a predilection for smooth and root surfaces that are usually resistant to decay in non-irradiated individuals (Palmier et al. 2020; Martins et al. 2021; Moore et al. 2020). Despite technological advancements such as intensity-modulated radiotherapy (IMRT), which enables highly conformal dose delivery and spares adjacent tissues, RRC remains prevalent (Duarte et al. 2014). This ongoing clinical burden underscores the need to revisit its pathogenesis beyond the traditional attribution to salivary gland dysfunction (Gupta et al. 2015).

Preventive strategies, including topical fluoride application, meticulous oral hygiene, dietary modifications, and frequent dental follow-up, are routinely implemented (Sroussi et al. 2017; Brennan et al. 2022). Nevertheless, RRC can develop within months after radiotherapy and often progresses aggressively. Structural degradation of tooth tissues, coupled with reduced salivary flow, complicates restorative efforts (Jasmer et al. 2020; Gan et al. 2024). From a patient perspective, RRC leads to pain, hypersensitivity, tooth loss, and impaired oral function—factors that reduce quality of life and nutritional intake, especially in post-cancer recovery (Çelik et al. 2024). For clinicians, RRC is frustratingly recurrent, frequently necessitating multiple restorations or extractions (de Amorim et al. 2021).

Traditionally, RRC has been explained by radiation-induced salivary gland dysfunction, which diminishes saliva’s buffering, cleansing, and antimicrobial properties (Gupta et al. 2015). However, reports of rapidly progressing lesions even in patients with preserved salivary flow challenge this view and suggest that ionizing radiation may directly impair dental hard tissues (Chen et al. 2020). In contrast, the jawbone is well known to suffer direct radiation damage, leading to osteoradionecrosis (ORN), for which interventions such as shielding and hyperbaric oxygen therapy are widely applied (Gupta et al. 2015; Owosho et al. 2017; Suenaga et al. 2018). Yet, despite the structural similarities between bone and dentin, such as their shared reliance on a collagen-mineral matrix, direct damage to dental tissues has not been sufficiently explored.

Previous reports have demonstrated that ionizing radiation can damage dental hard tissues. Enamel shows microcracks, reduced hardness, and increased acid solubility (Liang et al. 2016; Lu et al. 2019), while dentin exhibits structural weakening, collagen breakdown, and mineral loss (Gonçalves et al. 2014; Hegde et al. 2025). The collagen matrix in dentin acts as a scaffold to maintain the mineralized structure. When this scaffold is damaged, the tissue becomes more vulnerable to demineralization and acid attack (McGuire et al. 2014; Springer et al. 2005). However, most studies have evaluated only limited aspects of this damage. The present study comprehensively investigated the effects of therapeutic radiation on enamel and dentin by analyzing acid resistance, surface hardness, collagen integrity, and local pH, which together provide a clearer understanding of how radiation may directly contribute to radiation-related caries. The aim of this study was thus to elucidate the direct effects of therapeutic radiation on dental hard tissues under clinically relevant conditions, by evaluating both mineral degradation and collagen scaffold disruption.

## Materials and methods

Sample preparation: Six non-carious mandibular third molars, extracted for clinical reasons at the Department of Oral and Maxillofacial Surgery, Tohoku University Hospital, were collected following ethical approval and stored at − 80 °C until use. Each tooth was sectioned longitudinally into two halves using a water-cooled diamond saw to create paired control and irradiation samples. Each fragment was then placed into a separate vinyl pack filled with phosphate-buffered saline (PBS), sealed to exclude air, and stored at 4 °C until use in experiments.

Tooth preparation and irradiation protocol: Extracted non-carious mandibular third molars were longitudinally sectioned into two halves to create paired control and irradiated samples. Each fragment was placed with the cut dentin surface facing upward to ensure maximum exposure. Radiation was delivered using a clinical linear accelerator under conditions mimicking intensity‑modulated radiation therapy (IMRT), following two distinct protocols. In the single-dose protocol, samples received 20 Gy in one session. In the fractionated protocol, samples were exposed to 2 Gy/day, 5 days/week for 7 weeks (total 70 Gy), replicating a standard clinical IMRT regimen. To simulate soft tissue conditions, each specimen was sealed in a PBS-filled vinyl pack and positioned on a 1.0 cm‑thick water‑equivalent bolus, with two additional bolus layers placed above the sample. A source‑to‑target distance (STD) of 100 cm was used. A designated reference point near the buccal crown surface was prescribed to receive 100% of the dose, and isodose mapping confirmed that the enamel and dentin regions were fully encompassed within the therapeutic dose region.

Vickers microhardness testing: Vickers hardness numbers (VHN) were obtained using a microhardness tester (FM-ARS9000, Future-Tech Corp., Tokyo, Japan) located at the Graduate School of Dentistry, Tohoku University, based on previous method (Lu et al. 2019). Measurements were performed on the following regions: crown dentin (internal surface/cut section), crown enamel (outer surface) and root dentin (outer surface). Each region was measured at three points, and the values were averaged per region (N = 3).

Acid resistance (calcium elution assay): A 6-mm-diameter filter disc (antibiotic assay paper, ADVANTEC, Japan) soaked in 20 µL of lactic acid solution (pH 4.5) was placed on the surface of enamel or dentin for 10 min (Chien et al. 2016). The eluted solution was analyzed for calcium content using QuantiChrom Calcium Assay Kit (Bio Assay Systems, Hayward, CA, USA), then measured in accordance with the manufacturer’s instructions using Varioskan LUX reader (Thermo Fisher Scientific) at an absorbance of 610 nm (Martins et al. 2012). Measurements were performed on the following regions: crown dentin (internal surface/cut section), crown enamel (outer surface) and root dentin (outer surface). Each region was measured at three points, and the values were averaged per region. (N = 3).

Autofluorescence imaging: Collagen degradation was assessed via autofluorescence spectroscopy using a Varioskan LUX reader (Thermo Fisher Scientific). Samples were excited at 360 nm, and emission was recorded at 450 nm (Lambrecht and Mallol 2020; Czermak et al. 2019). Fluorescence intensity was analyzed using imaging software and compared between groups. (N = 3).

pH measurement: After irradiation, the pH of the sample surfaces was measured using a flat-surface microelectrode probe (Horiba LAQUA F-72, Japan). Three points were measured per sample and averaged (N = 3). Local pH measurement of dental hard tissue surfaces using microelectrodes has been described previously (Ratanaporncharoen et al. 2018).

Statistical analysis: All data are presented as mean ± standard error. Statistical comparisons between control and irradiated samples were performed using paired t-tests. A p-value less than 0.05 was considered statistically significant.

## Results

### Irradiation outcome and dosimetric verification

**Fig. 1:**
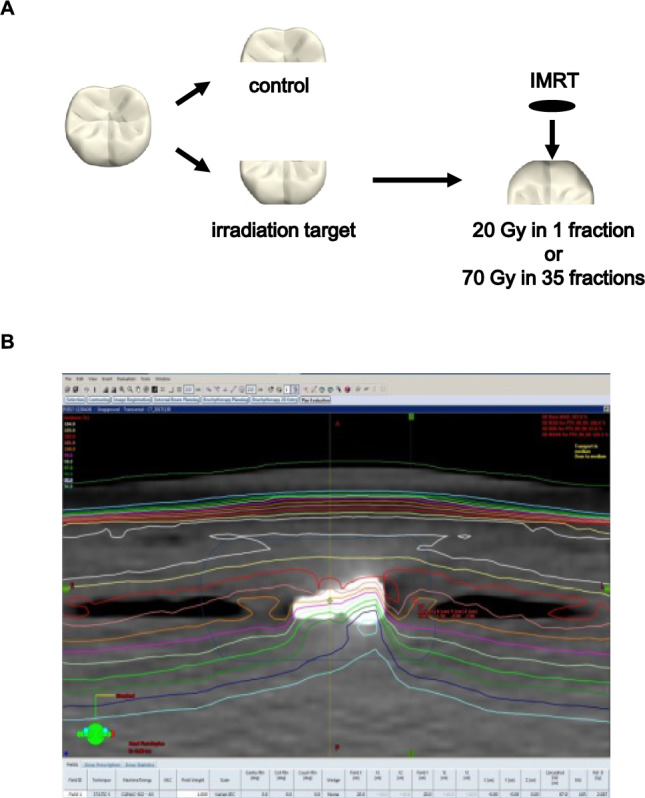
Tooth segmentation and IMRT-mimicking irradiation setup. (**A**) longitudinal sectioning of extracted molars showing defined enamel and dentin regions used for analyses. (**B**) experimental irradiation setup using a clinical linear accelerator with bolus layers to mimic IMRT conditions

Radiation was successfully delivered to all tooth specimens under clinically relevant protocols (20 Gy single dose or 70 Gy in 35 fractions). The segmentation and region definition used for enamel and dentin analyses are shown in Fig. 1A, and the IMRT-mimicking irradiation setup is shown in Fig. 1B. Dose verification confirmed that the targeted crown and dentin regions were fully encompassed within the therapeutic dose field (Fig. 1B), demonstrating the reproducibility and precision of the irradiation procedure.

**Fig. 2:**
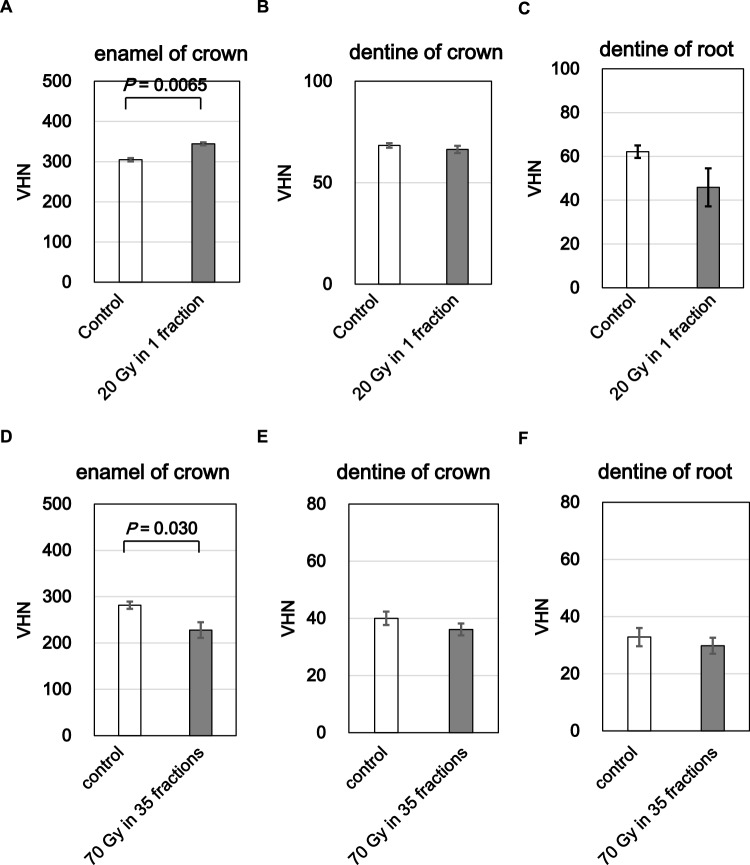
Vickers microhardness of enamel and dentin after irradiation. (**A**–**C**) hardness of crown enamel, crown dentin, and root dentin after a single 20 Gy dose. (**D**–**F**) corresponding hardness after 70 Gy in 35 fractions. irradiated regions showed reduced vickers hardness relative to paired controls. bars represent mean ± SE; significant p-values are indicated

### Changes in microhardness of enamel and dentin

To evaluate the mechanical integrity of dental hard tissues following irradiation, we measured microhardness in enamel and dentin. Vickers microhardness testing revealed a significant increase in hardness in crown enamel in the 20 Gy group compared to the control (p = 0.0065) (Fig. 2A). No significant changes were detected in crown dentin or root dentin (Fig. 2B, C). In contrast, the 70 Gy group exhibited a significant decrease in enamel hardness (p = 0.030) (Fig. 2D), while crown and root dentin values remained unchanged (Fig. 2E, F).

**Fig. 3:**
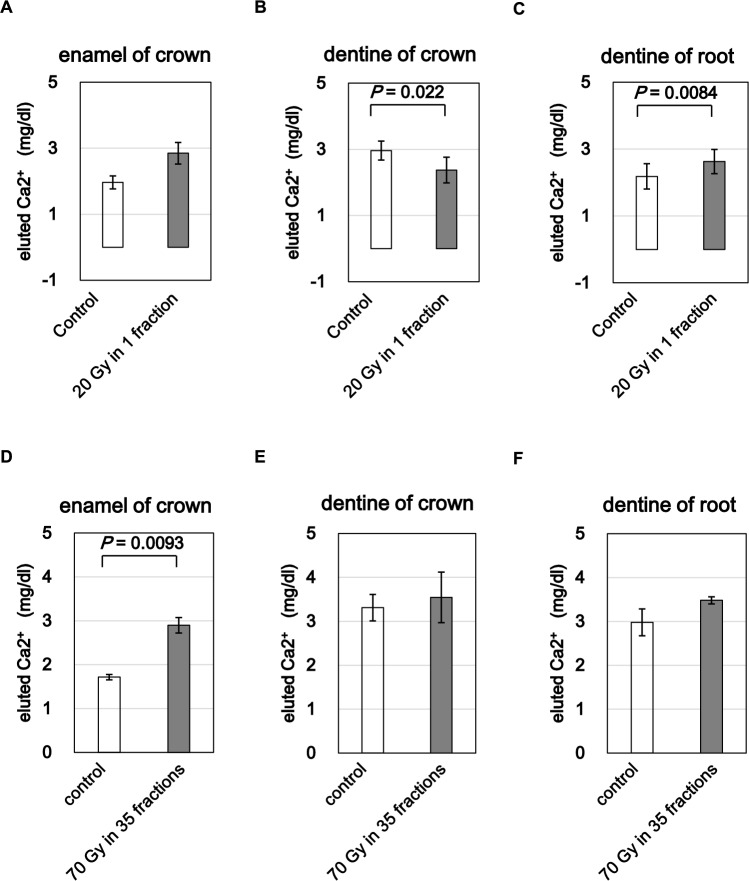
Acid resistance of enamel and dentin evaluated by calcium elution. (**A**–**C**) calcium release from crown enamel, crown dentin, and root dentin after 20 Gy. (**D**–**F**) corresponding release after 70 Gy in 35 fractions. irradiation increased calcium elution, indicating reduced acid resistance. bars represent mean ± SE; significant p-values are indicated

These findings suggest that radiation affects enamel hardness in a dose-pattern-dependent manner, with acute high-dose exposure inducing transient hardening and fractionated exposure resulting in demineralization. This may have implications for post-radiation enamel fragility under clinical conditions.

### Acid resistance of enamel and dentin

To assess chemical resistance, specifically the functional weakening of dental tissues following irradiation, we evaluated calcium elution after acid challenge. In the 20 Gy group, calcium release remained statistically unchanged in the enamel region (Fig. 3A), but a trend toward increased elution was observed, suggesting a reduction in acid resistance. Significant changes were detected in dentin: crown dentin showed reduced calcium release (p = 0.022) (Fig. 3B), while root dentin showed increased release (P = 0.0084) (Fig. 3C). In the 70 Gy group, a significant increase in calcium release was observed only in crown enamel (p = 0.0093) (Fig. 3D), with no significant differences in crown or root dentin (Fig. 3E,  F). These findings suggest that single high-dose irradiation may impair acid resistance in dentin, particularly root dentin, whereas fractionated exposure, more reflective of clinical IMRT primarily compromises enamel’s ability to resist demineralization.

**Fig. 4:**
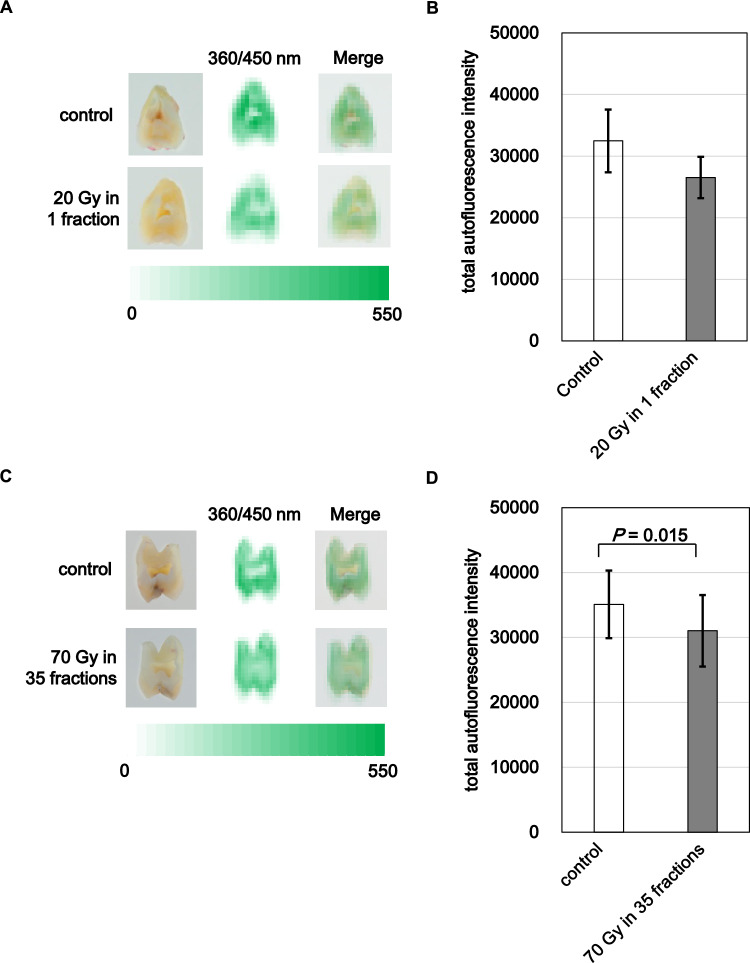
Autofluorescence imaging and collagen signal in irradiated dentin. (**A**, **C**) representative autofluorescence and merged images for 20 Gy and 70 Gy groups. (**B**, **D**) quantified total fluorescence intensities. irradiated dentin showed reduced collagen signal. bars represent mean ± SE; significant p-values are indicated

### Collagen degradation in dentin

Collagen, an organic component of dentin, provides the structural scaffold for mineral deposition. Its degradation or denaturation is expected to impair both the chemical and mechanical properties of teeth. Therefore, we assessed collagen integrity by autofluorescence imaging. Representative images of the internal cut surfaces showed a reduction in collagen-specific autofluorescence in irradiated samples. In the 20 Gy group, fluorescence intensity was decreased but not statistically significant (Fig. 4A, B). In contrast, the 70 Gy group exhibited a significant reduction in collagen signal (p = 0.015) (Fig. 4C, D). These results indicate that fractionated irradiation leads to cumulative collagen degradation, likely compromising the organic framework required for tooth mineral stability. These findings support the hypothesis that repeated low-dose irradiation causes progressive damage to the organic matrix, particularly collagen, while a single high-dose exposure induces a subtler effect. This collagen damage, which likely also occurs in the clinical setting, may destabilize the mineral scaffold of dentin, thereby contributing to the observed reduction in microhardness and acid resistance.

**Fig. 5:**
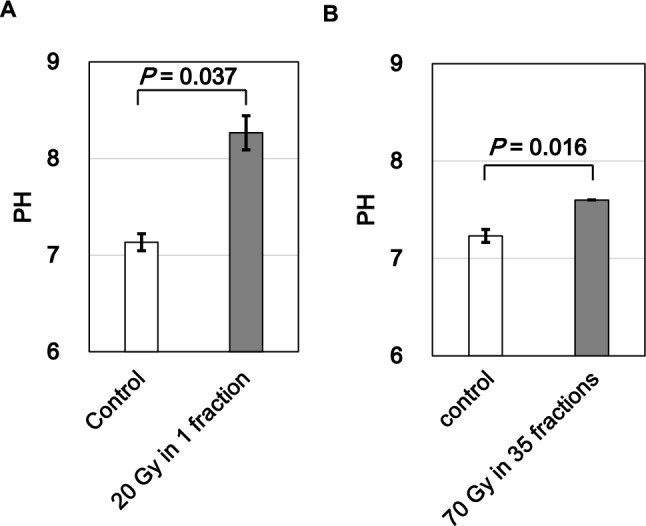
pH changes in dentin following therapeutic irradiation. (**A**) dentin pH after 20 Gy. (**B**) dentin pH after 70 Gy in 35 fractions. both irradiation regimens significantly increased dentin pH compared with paired controls. Bars represent mean ± SE; significant p-values are indicated

### Changes in internal pH of dentin

To explore possible environmental factors contributing to collagen degradation, we measured the internal pH of irradiated dentin. Measurements of the internal dentin surface revealed alkaline shifts in both irradiation groups. In the 20 Gy group, the pH increased significantly compared to control (p = 0.037) (Fig.  5A). A similar but more moderate increase was observed in the 70 Gy group (p = 0.016) (Fig. 5B). These results suggest that irradiation induces localized alkalinity, which may promote collagen breakdown and contribute to radiation-induced degradation of tooth structure. The larger pH shift observed after a single 20 Gy exposure may reflect the greater immediate ionizing effect of a high-dose fraction compared to fractionated dosing. This suggests that a single high dose may more acutely create an alkaline environment within dentin, potentially predisposing the organic matrix to damage. However, in our collagen analysis, only the 70 Gy group showed a significant reduction in collagen autofluorescence, while the 20 Gy group showed only a non-significant trend. This apparent discrepancy implies that collagen degradation is more strongly influenced by cumulative effects of repeated irradiation, rather than by transient physicochemical shifts alone. In other words, acute pH elevation may serve as a trigger, but repeated exposure likely drives structural deterioration of collagen over time, even with smaller individual effects. These findings suggest that distinct biological processes, instantaneous physicochemical stress and long-term matrix remodeling, both contribute to radiation-induced damage of dental organic tissues.

## Discussion

### Microhardness changes in enamel: comparison with previous findings

The presented results demonstrate a transient increase in enamel hardness after a single 20 Gy exposure, followed by decreased hardness under the clinically relevant 70 Gy fractionated protocol. This biphasic pattern is consistent with earlier reports that low or moderate dose radiation may temporarily increase enamel brittleness by reducing interprismatic spaces (Walker et al. 2011; Gonçalves et al. 2014), whereas higher cumulative doses promote demineralization and prism disruption (Liang et al. 2016; Chen et al. 2020). Thus, our findings align with the concept that enamel undergoes initial compaction followed by dose-dependent mineral breakdown, explaining its clinical susceptibility to chipping and cervical breakdown after radiotherapy.

### Altered acid resistance and calcium release

The increased calcium elution observed particularly in enamel at 70 Gy corroborates previous laboratory studies showing impaired resistance to acidic challenge after therapeutic radiation (Lopes et al. 2021; Gonçalves et al. 2014). Notably, our data extends these findings by demonstrating region-specific vulnerability: root dentin exhibited greater calcium release than crown dentin after a single 20 Gy exposure. This pattern is consistent with known structural differences in dentin, including higher tubular density and thinner peritubular mineral layers in root and cervical dentin (Komabayashi et al. 2008). Furthermore, irradiation has been reported to increase dentin permeability (Soares et al. 2009), which may further enhance susceptibility to demineralization in regions with inherently weaker microarchitecture. These results collectively suggest that radiation exacerbates pre-existing structural heterogeneity within the tooth, leading to region-dependent reductions in acid resistance.

### Collagen degradation: agreement with known effects on mineralized tissues

Collagen integrity was significantly compromised under fractionated irradiation, consistent with prior reports that ionizing radiation disrupts collagen cross-linking and reduces autofluorescence signal in bone and soft tissues (Springer et al. 2005; McGuire et al. 2014). Because dentin mineralization depends on a stable collagen scaffold, such degradation likely contributes to the observed reductions in microhardness and acid resistance. The present findings, therefore, provide direct evidence that collagen damage is a key mediator of radiation-induced dentin fragility, a mechanism previously suggested but not experimentally visualized in human dental tissue.

### pH elevation and physicochemical injury

A novel finding of this study is the significant increase in the internal pH of dentin after irradiation. Water radiolysis generates hydroxyl radicals (•OH) and related species (Spitz et al. 2004), and such reactions can create transient alkaline microenvironments. Alkaline conditions are known to destabilize collagen triple helices (Setoyama et al. 2024), suggesting a mechanistic link between physicochemical stress and subsequent matrix degradation. Interestingly, the 20 Gy group showed a larger acute pH elevation but milder collagen breakdown, whereas the 70 Gy group exhibited the opposite pattern, indicating that pH shifts act as an early trigger, while cumulative irradiation drives structural deterioration.

### Integration with the clinical presentation of RRC

Together, these data align well with clinical observations that RRC frequently affects enamel first (cervical enamel breakdown), that progresses rapidly and is resistant to conventional fluoride-based prevention (Brennan et al. 2022; Sroussi et al. 2017). The dual-mechanism model supported by our findings—salivary gland dysfunction combined with intrinsic weakening of enamel and dentin—helps explain why RRC can develop even when oral hygiene is adequate and fluoride therapy is provided.

### Clinical implications and prevention

Given that direct tooth damage contributes to RRC, approaches that physically shield teeth during radiotherapy warrant reconsideration. Custom mouthpieces or intraoral stents have been effective in reducing mucosal inflammation and osteoradionecrosis in brachytherapy (Suenaga et al. 2018), and similar devices could be adapted to protect dental tissues during external beam radiotherapy.

### Limitations

This study used extracted human molars isolated from oral environmental factors. In clinical settings, additional influences—xerostomia, mucositis, plaque biofilm, systemic therapy—interact with radiation effects. Thus, our findings should be interpreted as reflecting the direct physicochemical impact of irradiation, rather than the full multifactorial pathogenesis of RRC.

## Conclusion

The presented results show that therapeutic irradiation induces collagen degradation, mineral destabilization, reduced hardness, impaired acid resistance, and pH elevation, directly compromising the structural stability of enamel and dentin. This mechanistic insight clarifies why RRC can occur independent of saliva and highlights the importance of tooth-protective strategies during radiotherapy.

## Data Availability

All data supporting the findings of this study are contained within the manuscript.
